# Caffeine Therapy Reduces Severe Retinopathy of Prematurity in Neonates with Gestational Age between 23 and 28 Weeks

**DOI:** 10.1016/j.xops.2025.100903

**Published:** 2025-08-06

**Authors:** Yuki Otsuka, Futoshi Taketani, Miou Hirose, Hideyasu Oh

**Affiliations:** Department of Ophthalmology, Hyogo Prefectural Amagasaki General Medical Center, Amagasaki City, Hyogo, Japan

**Keywords:** Retinopathy of prematurity, Caffeine, Preterm neonate, Gestational age

## Abstract

**Purpose:**

To investigate the association of caffeine citrate therapy with the onset and exacerbation of retinopathy of prematurity (ROP).

**Design:**

A retrospective observational cohort study.

**Participants:**

Preterm neonates born at Hyogo Prefectural Amagasaki General Medical Center between January 2018 and December 2023 were included.

**Methods:**

Data on maternal (age; comorbid hypertensive disorders of pregnancy and gestational diabetes mellitus; fetal growth restriction; and cesarean section) and neonatal (sex; gestational age; birth weight; zone at initial examination; Apgar scores; respiratory distress syndrome; chronic lung disease; tracheal intubation; duration of tracheal intubation; duration of oxygen therapy; and caffeine therapy) risk factors for ROP were collected from medical records.

**Main Outcome Measures:**

Retinopathy of prematurity onset and severe ROP were defined as stage ≥1 and stage ≥3 during the course of follow-up, respectively. The association between caffeine therapy and the onset of any ROP and severe ROP was investigated.

**Results:**

Among 202 neonates included in the current study, 94 (46.5%) developed any ROP (stage ≥1) during the observational period. Only the zone at initial examination affected the onset of any ROP (*P* < 0.01). However, in 115 neonates born at <32 weeks gestational age and with <1500 g birth weight, larger zones at baseline and caffeine treatment were associated with decreased severe ROP (*P* = 0.017 and *P* < 0.01, respectively). Additionally, in 54 neonates with gestational age between 23 and 28 weeks, only caffeine therapy was associated with decreased development of severe ROP (*P* < 0.01). All 4 neonates with gestational age <23 weeks progressed to severe ROP despite caffeine therapy.

**Conclusions:**

Caffeine therapy may be a potential treatment strategy to prevent the progression of severe ROP in neonates born between 23 and 28 weeks' gestational age.

**Financial Disclosure(s):**

The authors have no proprietary or commercial interest in any materials discussed in this article.

Retinopathy of prematurity (ROP) is a vasoproliferative retinal disorder occurring in infants with low gestational age and birth weight. Technological advances in neonatal intensive care units and resuscitation have enabled the survival of infants with extremely low gestational age and birth weight. However, this has resulted in ROP becoming a major cause of childhood blindness worldwide.^1^, ^2^, ^3^ Retinopathy of prematurity is caused by oxygen-induced damage to the developing retinal vasculature, which leads to hyperoxia-induced vascular obliteration and abnormal growth of retinal blood vessels mediated by VEGF, insulin-like growth factor I, and other signaling pathways.^4^

Severe ROP is characterized by the development of retinal fibrovascular proliferation that leads to retinal detachment. Over the past decades, treatment for severe ROP has evolved from cryotherapy to laser photocoagulation targeting the peripheral avascular retina. Several randomized controlled trials have shown that anti-VEGF treatments, including bevacizumab^2^ ranibizumab,^5^ and aflibercept,^6^ have a robust effect on severe ROP. However, despite these treatments, ROP may still progress, requiring surgical intervention and eventually leading to total blindness. Therefore, it remains essential to explore novel therapies aimed at preventing ROP progression.

Various pharmacological interventions have the potential to prevent severe ROP, including caffeine citrate, antioxidants, cyclooxygenase inhibitors, human milk, and vitamins A and E.^7^ Although a randomized controlled trial suggested that caffeine treatment can lower the risk of severe ROP,^8^ its protective effect remains uncertain because some small retrospective studies showed no correlation.^9^^,^^10^ The purpose of this study was to investigate the effect of caffeine therapy on the onset and exacerbation of ROP.

## Methods

This single-center retrospective study was conducted at Hyogo Prefectural Amagasaki General Medical Center. The institutional review board and ethics committee of the hospital prospectively reviewed and approved the study protocol and waived the requirement for informed consent because of its retrospective nature. All study protocols adhered to the tenets of the Declaration of Helsinki.

### Study Subjects

We studied consecutive preterm neonates (gestational age <36 weeks or birth weight <3000 g) born at our hospital between January 2018 and December 2023 who were referred for ROP evaluation by the Department of Pediatrics. Neonates were excluded if they had already completed retinal vascularization at the time of first examination and required no further observations. We also excluded patients who were unable to undergo follow-up examinations because of transfer to another hospital.

The following data were collected from medical records: maternal age, maternal comorbidities, including hypertensive disorders of pregnancy and gestational diabetes mellitus, fetal growth restriction, cesarean section, sex, gestational age, birth weight, zone at initial examination, Apgar scores (1 and 5 minutes), respiratory distress syndrome (RDS), chronic lung disease, intraventricular hemorrhage, sepsis, blood transfusions, tracheal intubation, duration of tracheal intubation, duration of oxygen therapy, and caffeine therapy. In patients who underwent tracheotomy, duration of tracheal intubation was defined as the number of days until tracheotomy. Duration of oxygen therapy was defined as the hospitalization period if home oxygen therapy was introduced.

### Examinations for ROP

After the recommendation of the American Academy of Pediatrics regarding screening of preterm infants for ROP,^11^ initial screening was performed in 29 weeks for neonates with gestational age <27 weeks and 3 weeks after birth for neonates with gestational age ≥27 weeks. Screening examinations continued until complete retinal vascularization was confirmed. All neonates underwent indirect ophthalmoscopy performed by an ophthalmologist specializing in ROP. The infant's pupils were dilated with 1 drop of 0.5% phenylephrine and tropicamide (SANDOL P Ophthalmic Solution; SANTEN) at least 30 minutes before the examination. Zones and stages of patients were classified according to the *International Classification of Retinopathy of Prematurity, Third Edition*.^12^ Retinopathy of prematurity onset was defined as stage ≥1 during the course of follow-up. Patients who developed proliferation (stage ≥3) during the disease course were regarded as having exacerbation and defined as “severe ROP.” Fundus photography (RetCam 3, Clarity Medical Systems Inc) was performed at the discretion of the treating physician.

### Caffeine Therapy

Preterm infants received intravenous loading and maintenance doses of caffeine citrate (Respia; Nobelpharma) as determined by a pediatrician, either to prevent extubation failure or for the management of apnea of prematurity. The loading dose was 20 mg/kg of body weight, followed by a daily maintenance dose of 5 to 10 mg/kg, depending on the neonates' condition. In patients who required intermittent caffeine therapy, the duration of caffeine treatment was defined as the period up to the final day of its administration.

### Data Analyses

Data are expressed as mean ± standard deviation; *t* tests and χ2 tests were used to compare values between groups. All analyses were performed using R software, version 4.1.2 (R Core Team), and statistical significance was defined as *P* < 0.05.

## Results

A total of 202 neonates (105 males and 97 females) were included in this study. Table 1 summarizes the overall patient characteristics. Mean gestational age was 29.8 ± 3.5 weeks, and zone at initial examination was 2.3 ± 0.7. Tracheal intubation was performed in 136 patients (67.3%) and lasted for 15.1 ± 27.5 days. The mean duration of oxygen therapy was 54.3 ± 51.6 days. In total, 135 (66.8%) patients were started on caffeine citrate 11.8 ± 15.9 days after birth, which continued for 23.9 ± 18.6 days. Caffeine administration was initiated at extubation in 81 neonates (60.0%).

**Table 1:** Patient Characteristics

n (eyes)	202
Maternal age (yrs)	31.6 ± 6.7
Hypertensive disorders of pregnancy (%)	38 (18.8%)
Gestational diabetes mellitus (%)	12 (5.9%)
Fetal growth restriction (%)	26 (12.9%)
Cesarean section (%)	141 (69.8%)
Sex (male/female)	105/97
Gestational age (wks)	29.8 ± 3.5
Birth weight (grams)	1335.1 ± 545.3
Zone (initial examination)	2.3 ± 0.7
Apgar score (1 min)	4.9 ± 2.4
Apgar score (5 min)	7.0 ± 2.0
Respiratory distress syndrome (%)	103 (51.0%)
Chronic lung disease (%)	41 (20.3%)
Intraventricular hemorrhage (%)	16 (7.9%)
Sepsis (%)	35 (17.3%)
Blood transfusions (%)	45 (22.3%)
Tracheal intubation (%)	136 (67.3%)
Duration of tracheal intubation (days)	15.1 ± 27.5
Duration of oxygen therapy (days)	54.3 ± 51.6
Caffeine therapy (%)	135 (66.8%)

We first analyzed risk factors affecting the onset of any ROP (Table 2). Retinopathy of prematurity (stage ≥1) occurred in 94 (46.5%) neonates during the overall observational period. Univariate analysis showed that maternal and delivery-associated factors (maternal age, frequency of hypertensive disorders of pregnancy and gestational diabetes mellitus, fetal growth restriction, and cesarean section) were not significantly different between infants with or without ROP. In contrast, there were significant differences in nearly all infant-related factors, including gestational age, birth weight, zone at initial examination, Apgar scores, frequency of RDS, chronic lung disease, sepsis, blood transfusions, tracheal intubation, and the duration of tracheal intubation and oxygen therapy. However, there was no difference in the frequency of caffeine therapy (*P* = 0.81). Logistic regression analysis revealed that a small zone at initial examination was statistically significant in the onset of any ROP (Table 3).

**Table 2:** Univariate Analysis of Risk Factors Associated with Onset of Any ROP

	No ROP	ROP	*P* Value
n (eyes)	108	94	–
Maternal factor			
Maternal age (yrs)	31.1 ± 7.4	32.1 ± 5.8	0.32
HDP (%)	25 (23.1%)	13 (13.8%)	0.091
GDM (%)	7 (6.5%)	5 (5.3%)	0.73
Fetal growth restriction (%)	12 (11.1%)	14 (14.9%)	0.42
Caesarean section (%)	74 (68.5%)	67 (71.3%)	0.67
Infant factor			
Sex (male/female)	59/49	46/48	0.42
Gestational age (wks)	32.1 ± 2.6	27.8 ± 3.6	<0.01
Birth weight (grams)	1557.2 ± 404.3	1003.7 ± 481.7	<0.01
Zone (initial examination)	2.8 ± 0.4	1.8 ± 0.6	<0.01
Apgar score (1 min)	5.8 ± 2.2	3.8 ± 2.3	<0.01
Apgar score (5 min)	7.7 ± 1.4	6.2 ± 2.2	<0.01
RDS (%)	39 (36.1%)	64 (68.1%)	<0.01
Chronic lung disease (%)	11 (10.2%)	30 (31.9%)	<0.01
Intraventricular hemorrhage (%)	5 (4.6%)	10 (10.6%)	0.104
Sepsis (%)	11 (10.2%)	23 (24.5%)	<0.01
Blood transfusions (%)	10 (9.3%)	35 (37.2%)	<0.01
Tracheal intubation (%)	53 (49.1%)	83 (88.3%)	<0.01
Duration of tracheal intubation (days)	4.6 ± 13.5	27.1 ± 33.8	<0.01
Duration of oxygen therapy (days)	32.6 ± 35.1	79.2 ± 56.3	<0.01
Caffeine therapy (%)	73 (67.6%)	62 (66.0%)	0.81

GDM = gestational diabetes mellitus; HDP = hypertensive disorders of pregnancy; RDS = respiratory distress syndrome; ROP = retinopathy of prematurity.

**Table 3:** Logistic Regression Analysis of Risk Factors Associated with Onset of Any ROP

	No ROP	ROP	Risk Ratio (95% CI)	*P* Value
n (eyes)	108	94	–	–
Gestational age (wks)	32.1 ± 2.6	27.8 ± 3.6	0.76 (–0.61 to 0.042)	0.10
Birth weight (grams)	1557.2 ± 404.3	1003.7 ± 481.7	1.0 (–0.00096 to 0.0026)	0.36
Zone (initial examination)	2.8 ± 0.4	1.8 ± 0.6	0.08 (–3.8 to –1.5)	<0.01
Apgar score (1 min)	5.8 ± 2.2	3.8 ± 2.3	0.97 (–0.23 to 0.16)	0.74
RDS (%)	39 (36.1%)	64 (68.1%)	0.57 (–1.6 to 0.45)	0.29
Chronic lung disease (%)	11 (10.2%)	30 (31.9%)	0.53 (–1.8 to 0.48)	0.27
Sepsis (%)	11 (10.2%)	23 (24.5%)	1.81 (–0.52 to 1.7)	0.30
Blood transfusions (%)	10 (9.3%)	35 (37.2%)	1.26 (–0.85 to 1.3)	0.67
Tracheal intubation (%)	53 (49.1%)	83 (88.3%)	1.38 (–0.84 to 1.5)	0.58
Duration of oxygen therapy (days)	32.6 ± 35.1	79.2 ± 56.3	1.0 (–0.017 to 0.011)	0.50
Caffeine therapy (%)	73 (67.6%)	62 (66.0%)	0.60 (–1.39 to 0.34)	0.25

CI = confidence interval; RDS = respiratory distress syndrome; ROP = retinopathy of prematurity.

**Figure 1:**
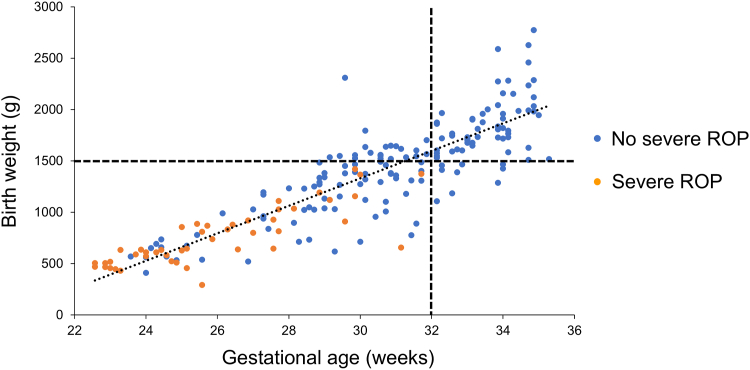
Development of severe ROP dependent on the gestational age or birth weight of neonates. The data from all 202 neonates were plotted. Horizontal and vertical axes indicate gestational age (weeks) and birth weight (grams), respectively. Blue points indicate patients who did not develop severe ROP, whereas orange points indicate those who developed proliferation. ROP = retinopathy of prematurity.

**Figure 2:**
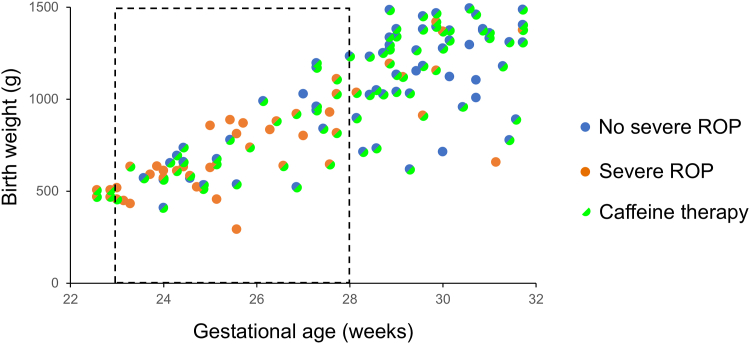
Development of severe ROP dependent on caffeine therapy. One hundred fifteen neonates with gestational age <32 weeks and birth weight <1500 g were plotted. Blue points indicate patients who did not develop severe ROP, whereas orange points indicate those who had proliferation. The points of patients who underwent caffeine therapy were split with green. The dot box includes patients with gestational age between 23 and 28 weeks. ROP = retinopathy of prematurity.

Next, we investigated risk factors related to the development of severe ROP. Because none of the infants with a gestational age >32 weeks or birth weight >1500 g developed severe ROP (Fig 1), 115 neonates with gestational age <32 weeks and birth weight <1500 g were examined to identify risk factors. Among these 115 neonates, 46 patients (40.0%) had a progression to severe ROP. Univariate analysis showed that along with the onset of any ROP, many factors (gestational age, birth weight, zone at initial examination, Apgar scores, frequency of RDS, sepsis, blood transfusions, tracheal intubation, and duration of tracheal intubation and oxygen therapy) were significantly associated with the incidence of severe ROP (Table 4). Interestingly, caffeine therapy was associated not only with the onset of any ROP but also with the development of severe ROP (*P* < 0.01). Logistic regression analysis showed that a larger zone at initial examination (*P* = 0.023, relative risk: 0.19, 95% confidence interval: –3.2 to –0.29) and caffeine therapy (*P* < 0.01, relative risk: 0.18, 95% confidence interval: –3.0 to –0.58) were significantly associated with decreased development of severe ROP (Table 5). Figure 2 shows whether each neonate with a gestational age <32 weeks and a birth weight <1500 g received caffeine therapy.

**Table 4:** Univariate Analysis of Risk Factors Associated with Severe ROP

	No Severe ROP	Severe ROP	*P* Value
n (eyes)	69	46	–
Maternal factor			
Maternal age (yrs)	32.2 ± 6.4	32.3 ± 5.86.2	0.93
HDP (%)	14 (20.3%)	8 (17.4%)	0.70
GDM (%)	3 (4.3%)	2 (4.3%)	0.96
Fetal growth restriction (%)	11 (15.9%)	9 (19.6%)	0.62
Caesarean section (%)	52 (75.4%)	34 (73.9%)	0.86
Infant factor			
Sex (male/female)	42/27	21/25	0.11
Gestational age (wks)	28.6 ± 2.3	25.9 ± 2.5	<0.01
Birth weight (grams)	1065.0 ± 303.3	775.2 ± 275.3	<0.01
Zone (initial examination)	2.1 ± 0.6	1.6 ± 0.4	<0.01
Stage (initial examination)	0.2 ± 0.4	0.4 ± 0.6	0.067
Apgar score (1 min)	4.2 ± 2.2	3.1 ± 1.9	<0.01
Apgar score (5 min)	6.7 ± 1.7	5.5 ± 2.3	<0.01
RDS (%)	40 (58.0%)	40 (87.0%)	<0.01
Chronic lung disease (%)	24 (34.8%)	14 (30.4%)	0.63
Intraventricular hemorrhage (%)	8 (11.6%)	4 (8.7%)	0.62
Sepsis (%)	8 (11.6%)	14 (30.4%)	0.012
Blood transfusions (%)	18 (26.1%)	22 (47.8%)	0.016
Tracheal intubation (%)	55 (79.7%)	45 (97.8%)	<0.01
Duration of tracheal intubation (days)	14.6 ± 19.6	41.7 ± 41.0	<0.01
Duration of oxygen therapy (days)	63.8 ± 37.0	107.3 ± 60.8	<0.01
Caffeine therapy (%)	60 (87.0%)	26 (56.5%)	<0.01

CI = confidence interval; GDM = gestational diabetes mellitus; HDP = hypertensive disorders of pregnancy; RDS = respiratory distress syndrome; ROP = retinopathy of prematurity.

**Table 5:** Logistic Regression Analysis of Risk Factors Associated with Severe ROP

	No Severe ROP	Severe ROP	Risk Ratio (95% CI)	*P* Value
n (eyes)	69	46	–	–
Gestational age (wks)	28.6 ± 2.3	25.9 ± 2.5	0.99 (–0.38 to 0.37)	0.97
Birth weight (grams)	1065.0 ± 303.3	775.2 ± 275.3	1.0 (–0.0024 to 0.0043)	0.61
Zone (initial examination)	2.1 ± 0.6	1.6 ± 0.4	0.19 (–3.2 to –0.29)	0.023
Apgar score (1 min)	4.2 ± 2.2	3.1 ± 1.9	0.96 (–0.29 to 0.21)	0.74
RDS (%)	40 (58.0%)	40 (87.0%)	2.08 (–0.60 to 2.2)	0.30
Sepsis (%)	8 (11.6%)	14 (30.4%)	1.69 (–0.83 to 1.86)	0.44
Blood transfusions (%)	18 (26.1%)	22 (47.8%)	0.88 (–1.35 to 1.0)	0.83
Tracheal intubation (%)	55 (79.7%)	45 (97.8%)	1.23 (–2.2 to 3.5)	0.88
Duration of oxygen therapy (days)	63.8 ± 37.0	107.3 ± 60.8	1.02 (0.00045–0.033)	0.052
Caffeine therapy (%)	60 (87.0%)	26 (56.5%)	0.18 (–3.0 to –0.58)	<0.01

CI = confidence interval; RDS = respiratory distress syndrome; ROP = retinopathy of prematurity.

**Figure 3:**
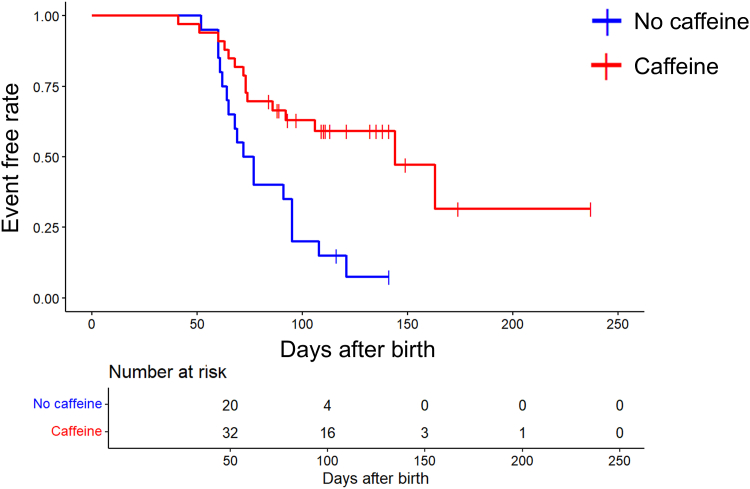
Kaplan–Meier analysis of severe ROP incidence in 54 neonates with gestational age between 23 and 28 weeks. A significant difference was found in the incidence rate of proliferation in neonates treated with caffeine (red line) and without caffeine (blue line) (*P* < 0.01). The log-rank test was used for statistical comparison. ROP = retinopathy of prematurity.

Additionally, we examined 54 neonates with gestational ages between 23 and 28 weeks to match the zone factor and clarify the effect of caffeine on severe ROP (Fig 2). Although there was no difference in zone at baseline in neonates with or without severe ROP (1.5 ± 0.5 vs. 1.6 ± 0.4; *P* = 0.35), caffeine was more frequently used for patients without severe ROP (*P* < 0.01) (Table 6). Logistic regression analysis showed that the incidence of severe ROP was only associated with caffeine therapy (*P* < 0.01, relative risk: 0.04, 95% confidence interval: –5.8 to –1.5) (Table 7). The initiation and duration of caffeine therapy were examined in patients who received caffeine in both groups. Caffeine therapy was initiated approximately 1 month after birth in both groups (with severe ROP: 31.5 ± 16.4 days and without severe ROP: 30.6 ± 18.4 days) and was continued for approximately 1 month (with severe ROP: 30.0 ± 19.0 days and without severe ROP: 31.6 ± 21.1 days). Neither the start time nor the duration of caffeine therapy differed significantly between the 2 groups (*P* = 0.88 and 0.82, respectively). The Kaplan–Meier curve compares the incidence of proliferation (severe ROP) between neonates treated with or without caffeine (Fig 3). A significant difference was found in 2 groups (*P* < 0.01). Meanwhile, 4 neonates with gestational age <23 weeks who were treated with caffeine therapy all progressed to severe ROP (Fig 2).

**Table 6:** Univariate Analysis of Risk Factors Associated with Severe ROP in Neonates with Gestational Age between 23 and 28 Wks

	No Severe ROP	Severe ROP	*P* Value
n (eyes)	21	33	–
Maternal factor			
Maternal age (yrs)	33.8 ± 6.4	32.4 ± 6.2	0.43
HDP (%)	3 (14.3%)	6 (18.2%)	0.71
GDM (%)	1 (4.8%)	2 (6.1%)	0.84
Fetal growth restriction (%)	2 (9.5%)	6 (18.2%)	0.38
Caesarean section (%)	15 (71.4%)	24 (72.7%)	0.92
Infant factor			
Sex (male/female)	12/9	17/16	0.69
Gestational age (wks)	25.7 ± 1.5	25.3 ± 1.5	0.34
Birth weight (grams)	774.5 ± 245.9	683.3 ± 190.0	0.13
Zone (initial examination)	1.6 ± 0.4	1.5 ± 0.5	0.35
Stage (initial examination)	0.1 ± 0.4	0.3 ± 0.6	0.19
Apgar score (1 min)	3.7 ± 2.0	3.1 ± 1.8	0.30
Apgar score (5 min)	5.8 ± 1.6	5.5 ± 2.0	0.51
RDS (%)	19 (90.5%)	30 (90.9%)	0.96
Chronic lung disease (%)	14 (66.7%)	13 (39.4%)	0.08
Intraventricular hemorrhage (%)	4 (19.0%)	4 (12.1%)	0.49
Sepsis (%)	5 (23.8%)	12 (36.4%)	0.33
Blood transfusions (%)	12 (57.1%)	19 (57.6%)	0.98
Tracheal intubation (%)	21 (100%)	33 (100%)	–
Duration of tracheal intubation (days)	35.9 ± 22.2	49.4 ± 44.0	0.2
Duration of oxygen therapy (days)	93.9 ± 30.2	117.0 ± 64.1	0.13
Caffeine therapy (%)	19 (90.5%)	14 (42.4%)	<0.01

GDM = gestational diabetes mellitus; HDP = hypertensive disorders of pregnancy; RDS = respiratory distress syndrome; ROP = retinopathy of prematurity.

**Table 7:** Logistic Regression Analysis of Risk Factors Associated with Severe ROP in Neonates with Gestational Age between 23 and 28 Wks

	No Severe ROP	Severe ROP	Risk Ratio (95% CI)	*P* Value
n (eyes)	21	33	–	–
Gestational age (wks)	25.7 ± 1.5	25.3 ± 1.5	2.57 (0.071–2.1)	0.055
Birth weight (grams)	774.5 ± 245.9	683.3 ± 190.0	1.0 (–0.071 to 0.0031)	0.47
Zone (initial examination)	1.6 ± 0.4	1.5 ± 0.5	0.19 (–4.0 to 0.40)	0.14
Apgar score (1 min)	3.7 ± 2.0	3.1 ± 1.8	1.0 (–0.41 to 0.41)	0.99
RDS (%)	19 (90.5%)	30 (90.9%)	1.48 (–2.5 to 3.2)	0.78
Sepsis (%)	5 (23.8%)	12 (36.4%)	4.15 (–0.4 to 3.5)	0.15
Duration of oxygen therapy (days)	93.9 ± 30.2	117.0 ± 64.1	1.03 (0.0010–0.058)	0.078
Caffeine therapy (%)	19 (90.5%)	14 (42.4%)	0.04 (–5.8 to –1.5)	<0.01

CI = confidence interval; RDS = respiratory distress syndrome; ROP = retinopathy of prematurity.

## Discussion

In this study, we investigated the association of caffeine citrate therapy with the development of any ROP and severe ROP. Both caffeine therapy and zone at baseline were significantly associated with decreased development of severe ROP in neonates with gestational age <32 weeks and birth weight <1500 g. Moreover, caffeine reduced the incidence of severe ROP independent of baseline zone, particularly in neonates with gestational age between 23 and 28 weeks.

Caffeine citrate was first demonstrated to be beneficial in treating apnea of prematurity in the 1970s.^13^ Caffeine is a type of methylxanthine and acts as a nonselective adenosine receptor antagonist that blocks the release of endogenous adenosine. Caffeine therapy mitigates hyperoxia-induced vaso-obliteration and hypoxia-induced angiogenesis through both adenosine A_2_A receptor-dependent and A_2_A receptor-independent mechanisms.^14^ Caffeine also has an indirect influence on dopaminergic activity by preventing the inhibitory effects of adenosine on dopamine receptors.^15^ In a prospective clinical phase III trial, caffeine therapy reduced severe ROP in preterm infants with apnea of prematurity compared with placebo control during a 2-year observation period.^8^^,^^16^ Although this can be most simply explained as secondary consequences of improving apnea symptoms, several underlying mechanisms have been described for the therapeutic potential of caffeine. For instance, caffeine modulates angiogenic factors such as hypoxia-inducible factor-1α, VEGF, sonic hedgehog, and matrix metalloproteinases, endothelial cell apoptosis, and oxidative stress.^17^, ^18^, ^19^, ^20^ Furthermore, caffeine may enhance the anti-inflammatory effects of cyclooxygenase inhibitors on activated microglia in the retina.^21^ Thus, caffeine appears to suppress retinal neovascular proliferation, but the exact mechanism remains unexplored.

As shown in a previous randomized controlled trial,^16^ our results demonstrated an association between caffeine therapy and decreased severe ROP. The time to proliferation was longer in neonates treated with caffeine than without, suggesting that caffeine administration may delay ROP progression. In addition to the therapeutic effect on apnea, caffeine is known to prevent extubation failure when administered at least 24 hours before a scheduled extubation.^22^ Furthermore, this effect was more pronounced in the stratified subgroup of infants with gestational age <28 weeks. In the present study, caffeine therapy was initiated at the time of extubation in more than half the cases, indicating that caffeine administration prevented the development of severe ROP by stabilizing respiratory status after extubation. Conversely, no other respiratory factors, including RDS incidence, the duration of tracheal intubation, and oxygen therapy, exhibited differences between neonates with and without severe ROP. Other pharmacological actions of caffeine discussed above may also be involved in preventing severe ROP.

It is widely known that ROP is a multifactorial disease, and its onset and exacerbation are influenced by a combination of diverse factors such as hyperoxemia, hypoxemia, substantial fluctuations in oxygen saturation, maternal or postnatal infection, transfusions, and poor postnatal weight gain.^23^^,^^24^ In the present study, neonates with gestational age >32 weeks or birth weight >1500 g did not develop severe ROP, whereas all 4 infants with gestational age <23 weeks progressed to severe ROP despite caffeine therapy. These results suggest that patients with a longer gestational age can be monitored without intervention and do not progress to proliferative disease, even if ROP occurs. Caffeine therapy alone was inadequate in preventing severe ROP in the most vulnerable cases with a short gestational age. One of the reasons why the effect of caffeine was obscure in previous studies could be explained by the fact that the population studies included too many stages of ROP. By limiting the population to those with gestational age between 23 and 28 weeks, we were able to identify a subgroup that may benefit most from caffeine therapy.

This study has some limitations, including its small sample size from a single hospital and its retrospective design. More studies, including a larger number of patients from multiple centers, are required to provide sufficient evidence of caffeine-induced effect. Lastly, caffeine citrate was not used for preventing ROP in the present study. Although there was no significant difference, early prophylactic administration tended to decrease severe ROP incidence compared with conventional therapy.^25^ Administration of therapeutic caffeine for ROP could be more effective in preventing severe ROP.

In conclusion, our study demonstrated that caffeine therapy may be a potential treatment for preventing the progression of severe ROP in neonates with gestational age <32 weeks and birth weight <1500 g, especially in those with gestational age between 23 and 28 weeks, irrespective of their baseline zone. Evaluation of optimal dosage and duration of caffeine administration is warranted in future studies.
